# Associations Between Procrastination and Subsequent Health Outcomes Among University Students in Sweden

**DOI:** 10.1001/jamanetworkopen.2022.49346

**Published:** 2023-01-04

**Authors:** Fred Johansson, Alexander Rozental, Klara Edlund, Pierre Côté, Tobias Sundberg, Clara Onell, Ann Rudman, Eva Skillgate

**Affiliations:** 1 Department of Health Promotion Science, Sophiahemmet University, Stockholm, Sweden; 2 Department of Psychology, Uppsala University, Uppsala, Sweden; 3 Department of Clinical Neuroscience, Karolinska Institutet, Stockholm, Sweden; 4 Unit of Intervention and Implementation Research for Worker Health, Institute of Environmental Medicine, Karolinska Institutet, Stockholm, Sweden; 5 Institute for Disability and Rehabilitation Research and Faculty of Health Sciences, Ontario Tech University, Oshawa, Ontario, Canada; 6 Department of Caring Sciences, Dalarna University, Falun, Sweden

## Abstract

**Importance:**

Procrastination is prevalent among university students and is hypothesized to lead to adverse health outcomes. Previous cross-sectional research suggests that procrastination is associated with mental and physical health outcomes, but longitudinal evidence is currently scarce.

**Objective:**

To evaluate the association between procrastination and subsequent health outcomes among university students in Sweden.

**Design, Setting, and Participants:**

This cohort study was based on the Sustainable University Life study, conducted between August 19, 2019, and December 15, 2021, in which university students recruited from 8 universities in the greater Stockholm area and Örebro were followed up at 5 time points over 1 year. The present study used data on 3525 students from 3 time points to assess whether procrastination was associated with worse health outcomes 9 months later.

**Exposure:**

Self-reported procrastination, measured using 5 items from the Swedish version of the Pure Procrastination Scale rated on a Likert scale from 1 (“very rarely or does not represent me”) to 5 (“very often or always represents me”) and summed to give a total procrastination score ranging from 5 to 25.

**Main Outcomes and Measures:**

Sixteen self-reported health outcomes were assessed at the 9-month follow-up. These included mental health problems (symptoms of depression, anxiety, and stress), disabling pain (neck and/or upper back, lower back, upper extremities, and lower extremities), unhealthy lifestyle behaviors (poor sleep quality, physical inactivity, tobacco use, cannabis use, alcohol use, and breakfast skipping), psychosocial health factors (loneliness and economic difficulties), and general health.

**Results:**

The study included 3525 participants (2229 women [63%]; mean [SD] age, 24.8 [6.2] years), with a follow-up rate of 73% (n = 2587) 9 months later. The mean (SD) procrastination score at baseline was 12.9 (5.4). An increase of 1 SD in procrastination was associated with higher mean symptom levels of depression (β, 0.13; 95% CI, 0.09-0.17), anxiety (β, 0.08; 95% CI, 0.04-0.12), and stress (β, 0.11; 95% CI, 0.08-0.15), and having disabling pain in the upper extremities (risk ratio [RR], 1.27; 95% CI, 1.14-1.42), poor sleep quality (RR, 1.09, 95% CI, 1.05-1.14), physical inactivity (RR, 1.07; 95% CI, 1.04-1.11), loneliness (RR, 1.07; 95% CI, 1.02-1.12), and economic difficulties (RR, 1.15, 95% CI, 1.02-1.30) at the 9-month follow-up, after controlling for a large set of potential confounders.

**Conclusions and Relevance:**

This cohort study of Swedish university students suggests that procrastination is associated with subsequent mental health problems, disabling pain, unhealthy lifestyle behaviors, and worse psychosocial health factors. Considering that procrastination is prevalent among university students, these findings may be of importance to enhance the understanding of students’ health.

## Key Points

**Question:**

Is procrastination associated with subsequent health outcomes among university students?

**Findings:**

In this cohort study of 3525 Swedish university students, procrastination was associated with worse subsequent mental health (depression, anxiety, and stress symptom levels), having disabling pain in the upper extremities, unhealthy lifestyle behaviors (poor sleep quality and physical inactivity), and worse levels of psychosocial health factors (higher loneliness and more economic difficulties).

**Meaning:**

This study suggests that procrastination may be associated with a range of health outcomes.

## Teaser

This cohort study evaluates the association between procrastination and subsequent health outcomes among university students in Sweden.

## Introduction

Procrastination is defined as voluntarily delaying an intended course of action despite expecting to be worse off because of the delay^1^ and is common, especially among younger people.^2,3^ It is estimated that at least half of university students engage in consistent and problematic procrastination, such as postponing studying for examinations or writing papers.^1,4^ Procrastination is described as a form of self-regulatory failure linked to personality traits such as impulsiveness,^5,6^ distractibility, and low conscientiousness.^1,7^ An individual’s tendency to procrastinate is relatively stable over time, but specific procrastination behaviors are influenced by contextual factors such as task aversiveness.^1^ For some students, procrastination is occasional and related to specific academic tasks, while for others it is more of a general disposition, potentially affecting academic achievements^8^ and health.

Cross-sectional studies suggest that procrastination is associated with symptoms of depression, anxiety, and stress as well as loneliness and reduced life satisfaction.^2,9,10,11^ Procrastination is also associated with prevalent general physical health problems,^12^ cardiovascular disease,^13^ and unhealthy lifestyle behaviors.^12,14,15^

Students engaged in university studies have high levels of freedom and low structure, which places high demands on their capacity to self-regulate.^16^ These high demands on self-regulation may explain the high prevalence of procrastination among university students and make persons who are prone to procrastinate more vulnerable to the negative consequences of procrastination while at the university.^16^

The procrastination health model^12,17^ suggests that a general tendency to procrastinate is associated with negative health outcomes by increasing levels of stress, reducing healthy behaviors, and delaying treatment.^12,17,18^ However, the causal direction between procrastination and health outcomes is not well understood and could be bidirectional, with poor physical or mental health reducing energy levels and motivation, potentially leading to more procrastination.

Some of the strongest support for the causal claims of the procrastination health model comes from studies on the effects of treating procrastination. Results from randomized clinical trials suggest that intervening on procrastination with psychological treatments can reduce subsequent levels of depression and anxiety and improve quality of life.^19^ However, the evidence is limited as there are only a few studies, with a focus solely on mental health outcomes.^19^

The procrastination health model provides a useful theoretical foundation for how procrastination could affect different health outcomes.^12,17^ However, longitudinal evidence from studies of procrastination and subsequent health outcomes is scant and focuses on only 1 or a few health outcomes.^20,21,22,23^ Furthermore, most studies have limited control for important confounders, including other health problems, age, and sociodemographic factors. Also, no study has, to our knowledge, controlled for the potential reverse causation that may arise if health problems increase procrastination.

In this study, we investigated the associations between procrastination and a range of subsequent health outcomes while controlling for a large set of potential confounders, including prior levels of the outcomes. This method allows for stronger conclusions regarding the causal direction^24^ between procrastination and health outcomes and can provide a broader picture of the potential associations between procrastination and health.

We aimed to evaluate the associations between procrastination and 16 health outcomes at the end of a 9-month period. The outcomes include mental health, disabling pain, and several unhealthy lifestyle behaviors and psychosocial health factors. Following the procrastination health model,^12,17^ we hypothesized that higher levels of procrastination would be associated with worse health outcomes at follow-up.

## Methods

### Design and Study Population

The cohort for this study came from the Sustainable University Life study,^25^ which followed up Swedish university students for 1 year using web-based surveys. Undergraduate or graduate students (up to and including masters’ level) at full-time educational programs, with at least 1 year left of their education were eligible to participate. Students were recruited from 8 universities in the greater Stockholm area and Örebro. Data collection was ongoing from August 19, 2019, to December 15, 2021. The targeted universities represent a convenience sample and were selected to provide a variety of different types of disciplines, such as medicine, technology, social sciences, and economics and were restricted to universities in a limited geographical area to enable physical presence by study staff. The study was approved by the Swedish Ethical Review Authority and all participants provided informed consent electronically. More information about the study methods and data collection is available in the study protocol.^25^ This study is reported following the Strengthening the Reporting of Observational Studies in Epidemiology (STROBE) reporting guideline for cohort studies.

**Figure 1.**
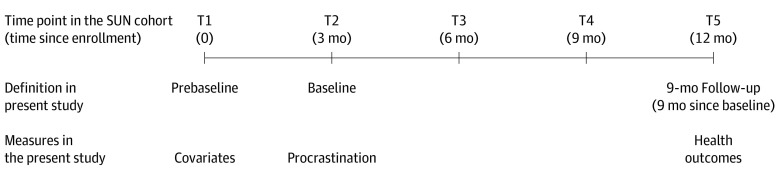
Timeline of Data Collection in the Sustainable University Life (SUN) Cohort and How Data Were Organized for the Present Study T indicates time point.

**Figure 2.**
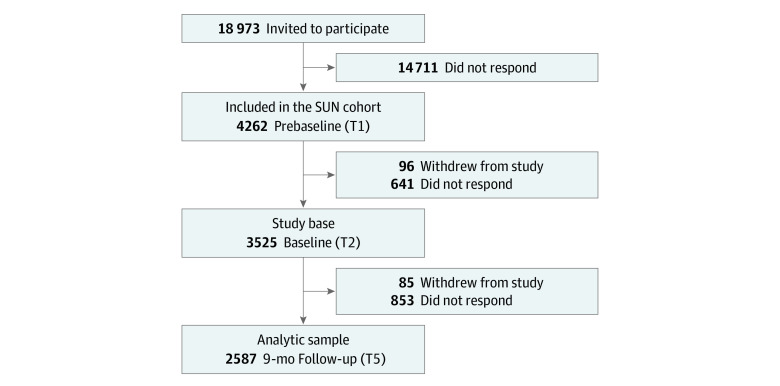
Flowchart of the Inclusion of Participants The definition of each time point (T) in the present study (T1, T2, and T5) refers to the time point in the Sustainable University Life (SUN) Cohort (Figure 1). Note that T3 and T4 are not shown here, because data from these time points were not used for the main analyses.

Eligible students were informed about the study during an in-class presentation and/or by an email with a link to the survey. Students who agreed to participate were followed up every 3 months for 1 year at 5 time points. In the present data analysis, the first time point was defined as prebaseline and was used for measures of the covariates. The second time point was defined as the baseline and was used for the procrastination measure and the fifth time point was defined as the 9-month follow-up and used for measurement of the health outcomes (Figure 1). The follow-up period of 9 months represents the length of an academic year, which we believe is adequate for procrastination to manifest its potential associations with different health outcomes. The sample was restricted to those responding at baseline and included 3525 participants; the follow-up rate was 73% (n = 2587) at the 9-month follow-up (Figure 2).

### Measures

#### Exposure: Procrastination

Procrastination was measured using 5 items from the Swedish version of the Pure Procrastination Scale (PPS).^26^ The items were rated on a Likert scale from 1 (“very rarely or does not represent me”) to 5 (“very often or always represents me”) and summed to give a total procrastination score ranging from 5 to 25. The short version of the PPS includes items 4 through 8 from the full PPS and has shown adequate psychometric properties in nonclinical samples.^26,27^ In this sample, the scale had a Cronbach α of 0.92 at baseline and a test-retest reliability (Pearson *r*) of 0.79 over 3 months and of 0.75 over 9 months.

#### Outcomes

We assessed a range of health outcomes at the 9-month follow-up, including mental health problems (symptoms of depression, anxiety, and stress), disabling pain (neck and/or upper back, lower back, upper extremities, and lower extremities), unhealthy lifestyle behaviors (poor sleep quality, physical inactivity, weekly tobacco use, monthly cannabis use, daily alcohol use, and breakfast skipping), psychosocial health factors (loneliness and economic difficulties), and general health. For more information on the outcome measures, see eMethods 1 in Supplement 1.

#### Confounders

Potential confounders were selected using the modified disjunctive cause criterion.^28^ This approach is a feasible alternative to directed acyclic graphs when the causal knowledge needed to produce a reliable directed acyclic graph is unavailable.^28^ We used the same set of covariates in all the outcome models, as has been suggested for outcome-wide studies.^29^ The covariate set included all outcome variables at prebaseline to limit the risk of reverse causation,^30^ as well as age, gender, highest parental level of education, previous physical and psychiatric diagnoses, civil status, place of birth, and university education type. All covariates were measured at prebaseline to ensure that none were mediators between procrastination and the outcomes. Information on covariate measurement and coding is provided in eMethods 1 in Supplement 1.

### Statistical Analysis

The characteristics of the sample are presented in Table 1, both for the full sample and across quartiles of procrastination (procrastination score range, 5-25; first quartile, 5-9; second quartile, 10-12; third quartile, 13-17; and fourth quartile, 18-25). To assess whether procrastination was associated with the health outcomes at follow-up, we used the outcome-wide approach,^29^ a newly developed analytic approach to study associations between an exposure and multiple outcomes. We built separate multivariable regression models for each of the outcomes to assess the association between baseline procrastination and the different outcomes at the 9-month follow-up. For continuous outcomes, linear regression models were used. For binary outcomes, modified Poisson regressions^31^ were used (rather than logistic regression models because some outcomes were relatively common).

**Table 1.** Characteristics of Participants Prebaseline for the Full Sample and by Quartiles of Procrastination Levels^a^

Characteristic	Participants, No. (%)				
	Full sample (N = 3525)	Procrastination quartile^b^			
		First (n = 1052)	Second (n = 713)	Third (n = 1003)	Fouth (n = 757)
Age, mean (SD), y	24.8 (6.2)	25.3 (6.7)	24.8 (6.3)	24.7 (6.2)	24.0 (5.5)
Gender					
Female	2229 (63)	674 (64)	458 (64)	640 (64)	457 (60)
Male	1274 (36)	NR^c^	NR^c^	NR^c^	NR^c^
Other	22 (1)	NR^c^	NR^c^	NR^c^	NR^c^
Education type					
Medical or health	1688 (48)	556 (53)	343 (48)	471 (47)	318 (42)
Technical	1407 (40)	354 (34)	274 (38)	413 (41)	366 (48)
Social science or humanities	294 (8)	100 (10)	68 (10)	79 (8)	47 (6)
Economic	85 (2)	28 (3)	16 (2)	28 (3)	13 (2)
Other	51 (1)	14 (1)	12 (2)	12 (1)	13 (2)
Civil status					
Single	1975 (56)	494 (47)	395 (55)	594 (59)	492 (65)
Cohabiting partner	1133 (32)	435 (41)	227 (32)	294 (29)	177 (23)
Noncohabiting partner	417 (12)	123 (12)	91 (13)	115 (11)	88 (12)
Place of birth					
Sweden	2826 (80)	859 (82)	579 (81)	797 (79)	591 (78)
Other Nordic countries	118 (3)	38 (4)	23 (3)	31 (3)	26 (3)
Other Europe	192 (5)	57 (5)	41 (6)	61 (6)	33 (4)
Outside Europe	389 (11)	98 (9)	70 (10)	114 (11)	107 (14)
Highest parental educational level					
University	2553 (72)	747 (71)	513 (72)	737 (73)	556 (73)
Less than university	972 (28)	305 (29)	200 (28)	266 (27)	201 (27)
Previous diagnoses, mean (SD), No.^d^	1.0 (1.3)	0.9 (1.2)	1.0 (1.3)	1.1 (1.2)	1.2 (1.4)
Mental health score, mean (SD)					
Depression symptoms	4.7 (4.7)	2.9 (3.6)	4.1 (4.2)	5.0 (4.4)	7.3 (5.4)
Anxiety symptoms	3.0 (3.3)	2.1 (2.9)	2.7 (3.1)	3.2 (3.2)	4.2 (4)
Stress symptoms	6.3 (4.6)	5.0 (4.3)	6.0 (4.3)	6.7 (4.4)	7.9 (4.8)
Disabling pain					
Neck and/or upper back	405 (11)	90 (9)	84 (12)	117 (12)	114 (15)
Lower back	364 (10)	86 (8)	62 (9)	117 (12)	99 (13)
Upper extremities	436 (12)	90 (9)	98 (14)	137 (14)	111 (15)
Lower extremities	545 (15)	123 (12)	106 (15)	162 (16)	154 (20)
Lifestyle behaviors					
Poor sleep quality	1893 (54)	380 (36)	351 (49)	605 (60)	557 (74)
Physically inactive	1831 (52)	438 (42)	364 (51)	548 (55)	481 (64)
Daily alcohol use	51 (1)	7 (1)	17 (2)	12 (1)	15 (2)
Weekly tobacco use	542 (15)	136 (13)	130 (18)	141 (14)	135 (18)
Monthly cannabis use	72 (2)	12 (1)	17 (2)	16 (2)	27 (4)
Breakfast skipping	878 (25)	188 (18)	160 (22)	246 (25)	284 (38)
Psychosocial health factors					
Loneliness	1485 (42)	273 (26)	275 (39)	477 (48)	460 (61)
Economic difficulties	555 (16)	110 (10)	110 (15)	172 (17)	163 (22)
Poor general health	113 (3)	14 (1)	11 (2)	34 (3)	54 (7)

Abbreviation: NR, not reported.

^a^ Procrastination was measured at baseline, while all covariates and outcomes were measured prebaseline. Percentages of some categorical variables do not add up to 100% due to rounding.

^b^ The procrastination score ranged from 5 to 25, with the quartiles 9 (25th percentile), 12 (50th percentile), and 17 (75th percentile). Participants were categorized based on their procrastination score as follows: 5 to 9, first quartile; 10 to 12, second quartile; 13 to 17, third quartile; and 18 to 25, fourth quartile.

^c^ The gender categories “male” and “other” are not presented for procrastination quartiles because the “other” category had low cell counts (<5). The gender category “other” included participants responding “Other” or “Do not want to identify with either of these identities.”

^d^ The previous diagnoses variable is a count indicating how many of the following diagnoses the participants had received: rheumatic diseases; allergies; respiratory diseases; cardiovascular diseases; gastrointestinal diseases; diabetes; diseases in urinary tracts or internal or external genitals; diseases in the nervous system, eyes, or ears; mental disorders; tumors or cancers; and attention deficits or learning disabilities.

Procrastination and all continuous outcome measures were standardized (mean = 0, SD = 1). Estimates for continuous outcomes are thus interpreted as the difference in the outcome as measured in SDs associated with a 1-SD increase in procrastination, and estimates for the binary outcomes are interpreted as the risk ratio (RR) of the outcome associated with a 1-SD increase in procrastination. All models were adjusted for the set of prebaseline confounders described. For unadjusted estimates, see eTable 1 in Supplement 1. Visual inspection of the residuals from all models indicated fairly linear associations between procrastination and the outcomes. The homoscedasticity and distribution of the residuals were not assessed because they typically have minor impact on the regression estimates,^32^ but the modified Poisson models still applies robust SEs. This analytic strategy deviates from the ones prespecified in our study protocol^25^ but was selected as it was deemed most appropriate for the research questions. Analyses were conducted using R, version 4.1.2 (R Group for Statistical Computing).

#### Missing Data

Outcome data were missing for 938 participants not responding at the 9-month follow-up, and the main analyses were performed as complete-case analyses (n = 2587). Prebaseline characteristics stratified by missingness at the 9-month follow-up are presented in eTable 2 in Supplement 1. We had complete data on the exposure and all covariates, except for the sleep quality variable. The Pittsburgh Sleep Quality Index (PSQI) had missing data on 3 items for sleep disturbances for 9% (n = 225) of the respondents in the analytic sample due to a technical error at the beginning of data collection. These missing data were handled by imputing the person-mean of the items for sleep disturbances.

#### Sensitivity Analyses

First, we computed the E-value^33,34^ that assesses how strongly unmeasured and residual confounding would need to be associated with the exposure and the outcomes on the RR scale to reduce the point estimates to the null. When the 95% CIs did not include the null, we also computed the E-value of the 95% CI bound closest to the null, to assess the strength of unmeasured and residual confounding needed to shift the 95% CI to include the null. Second, we calculated the point-biserial correlation between procrastination at baseline and missingness at follow-up to investigate the possibility of selection bias in relation to the internal validity. No association between the exposure and loss to follow-up indicates that selection bias cannot affect the internal validity, but only the external validity.^35^ Third, we conducted analyses controlling for prior levels of procrastination, as has been suggested for outcome-wide studies.^29^ These analyses had to be performed with a 6-month follow-up because we did not have data on procrastination at prebaseline (eMethods 2 and eTable 3 in Supplement 1). Fourth, the robustness of our results to the person-mean imputation of values on the PSQI was assessed by performing multiple imputation for the 3 missing items (eMethods 3 in Supplement 1).

## Results

At prebaseline, the study included 3525 participants (2229 women [63%] and 1274 men (36%); mean [SD] age, 24.8 [6.2] years) (Table 1). The mean (SD) procrastination score at baseline was 12.9 (5.4). Gender and age were similar across levels of procrastination, but participants with higher levels of procrastination tended to be more likely to study technical sciences, be single, and have been born outside of Europe. All outcome variables showed higher prevalence or mean levels at prebaseline among participants with higher procrastination levels at baseline.

Table 2 presents the results from the outcome regression models. At the 9-month follow-up, a 1-SD increase in procrastination was associated with higher mean symptom levels of depression (β, 0.13; 95% CI, 0.09-0.17), anxiety (β, 0.08; 95% CI, 0.04-0.12), and stress (β, 0.11; 95% CI, 0.08-0.15) as well as having disabling pain in the upper extremities (RR, 1.27; 95% CI, 1.14-1.42), poor sleep quality (RR, 1.09, 95% CI, 1.05-1.14), physical inactivity (RR, 1.07; 95% CI, 1.04-1.11), loneliness (RR, 1.07; 95% CI, 1.02-1.12), and economic difficulties (RR, 1.15, 95% CI, 1.02-1.30). The results showed no clear evidence of associations between procrastination and subsequent disabling pain in other body regions; use of tobacco, alcohol, or cannabis, breakfast skipping; or general health afer adjustment for the prebaseline covariates.

**Table 2.** Procrastination and Subsequent Health Outcomes^a^

Outcome	β or RR (95% CI)	E-value	
		PE	LCL
Mental health, β (95% CI)			
Depression symptoms	0.13 (0.09-0.17)	1.57	1.45
Anxiety symptoms	0.08 (0.04-0.12)	1.41	1.27
Stress symptoms	0.11 (0.08-0.15)	1.53	1.41
Disabling pain, RR (95% CI)			
Neck and/or upper back	1.09 (0.96-1.24)	1.41	NA
Lower back	1.03 (0.90-1.18)	1.22	NA
Upper extremities	1.27 (1.14-1.42)	1.86	1.53
Lower extremities	1.10 (0.99 to 1.23)	1.44	NA
Lifestyle behaviors, RR (95% CI)			
Poor sleep quality	1.09 (1.05-1.14)	1.41	1.27
Physical inactivity	1.07 (1.04-1.11)	1.35	1.24
Daily alcohol use	1.05 (0.71-1.56)	1.29	NA
Weekly tobacco use	0.96 (0.89-1.04)	1.24	NA
Monthly cannabis use	1.21 (0.91-1.62)	1.71	NA
Breakfast skipping	1.03 (0.97-1.09)	1.19	NA
Psychosocial health factors, RR (95% CI)			
Loneliness	1.07 (1.02-1.12)	1.35	1.17
Economic difficulties	1.15 (1.02-1.30)	1.57	1.17
Poor general health	1.14 (0.97-1.34)	1.54	NA

Abbreviations: LCL, lower confidence limit; NA, not applicable; PE, point estimate; RR, relative risk.

^a^ All models were adjusted for the set of prebaseline covariates described, including all outcomes prebaseline. Procrastination and all continuous outcomes were standardized (mean = 0 and SD = 1), and β is the standardized effect size.

For estimates with a 95% CI that excluded the null, the E-values suggested that unmeasured confounders would need to increase the risk of both procrastination and the outcomes by 35% to 86%, depending on the outcome, to move the point estimates to the null (Table 2). The point-biserial correlation between procrastination at baseline and missingness at the follow-up was 0.05. Outcome levels at prebaseline were generally somewhat higher in the group that was missing at the 9-month follow-up (eTable 2 in Supplement 1). The associations between procrastination and health outcomes 6 months later, while controlling for prior procrastination levels, were similar to the results in the main analysis (eMethods 2 and eTable 3 in Supplement 1). Using multiple imputation for the 3 missing items on the PSQI gave results identical to the main analysis when rounded to the second decimal place (eMethods 3 in Supplement 1).

## Discussion

In this cohort of Swedish university students, higher levels of procrastination were associated with worse subsequent mental health (depression, anxiety, and stress symptom levels), having disabling pain in the upper extremities, unhealthy lifestyle behaviors (poor sleep quality and physical inactivity), and worse levels of psychosocial health factors (higher loneliness and more economic difficulties) 9 months later. We found no clear associations between procrastination and subsequent disabling pain in other body regions (neck and/or upper back, lower back, or lower extremities), other unhealthy lifestyle behaviors (alcohol, tobacco, or cannabis use and breakfast skipping), or general health.

The identified associations are in the same direction, but to a lesser magnitude, than those reported in most previous studies on procrastination and health outcomes.^1,2,9,10,12^ There are several potential reasons for this difference in magnitude, such as the use of different scales to measure procrastination and the health outcomes. However, the arguably largest difference between this study and most previous studies is that we controlled for an extensive set of potential confounders. To our knowledge, this is the first study of procrastination and health outcomes to adjust for prior levels of the outcomes. As shown in eTable 1 in Supplement 1, when controlling for prior levels of the outcome variables, the unadjusted estimates of associations between procrastination and the health outcomes corresponded more closely to those of some previous studies.^2,9,10^ By adjusting for prebaseline levels of the outcome variables, we reduced the risk of reverse causality.^30^ Still, interpreting these association as causal effects requires the assumptions of no unmeasured or residual confounding, selection bias, or other biases affecting the associations,^36^ assumptions that are unlikely to be fully met in the present study. We do believe, however, that our results may correspond more closely to the consequences that interventions targeting procrastination would have for different health outcomes, than those of earlier cross-sectional studies.

Our estimates indicate that the associations between procrastination and subsequent health outcomes are weak. For instance, a 1-SD increase in procrastination was associated with a mean increase in subsequent depression symptoms of only 0.13 SDs, and many of the associations were weaker than this. The follow-up time of 9 months represents 1 academic year and was chosen because we believe this is sufficient induction time for procrastination to lead to health problems. It is possible, however, that these estimates would be stronger for a longer follow-up because the potential negative associations of procrastination with health outcomes could accumulate over time. Still, even though the associations are not very strong, it seems that procrastination could have associations with many different aspects of health, including mental health, physical pain, lifestyle behaviors, and psychosocial health factors. Thus, although it seems that intervening on procrastination is unlikely to produce large associations with any specific health outcome, it could possibly produce small associations with a diverse set of different health outcomes.

### Limitations

This study has some limitations. The E-value analysis suggests that unmeasured and residual confounding (from, for instance, neuroticism, conscientiousness, or genetic factors) could potentially explain away the observed associations. This potential unmeasured or residual confounding would, however, need to be moderately associated with procrastination and the outcomes, independent of all measured covariates^29^ (ie, associated with procrastination and the outcomes even after adjusting for all covariates). Given the large number of confounders adjusted for in these analyses, it is likely that a substantial proportion of any unmeasured confounding is already accounted for in our analyses. Controlling for prior procrastination levels gave results similar to those in the main analysis, limiting the risk of reverse causality and unmeasured confounding^29^ (eMethods 2 and eTable 3 in Supplement 1).

We also have the risk of misclassification of exposure and outcomes, which is probably not an issue for the procrastination measure given that the PPS showed excellent reliability. The outcomes, however, differ somewhat in their psychometric properties, and misclassification may have attenuated the observed associations for some of the outcomes.

We found that procrastination at baseline had a near-null association with loss to follow-up, so it is unlikely that the internal validity of observed associations is subject to selection bias.^35^ There were, however, small differences in prebaseline characteristics, especially prior outcome levels, between responders and nonresponders, which could affect external validity (eTable 2 in Supplement 1). Furthermore, the sample is not fully representative of the overall Swedish student population (for instance, we have more medical and health and technical students than average). Thus, it is uncertain whether our estimates are generalizable to Swedish students overall or to other populations. Furthermore, our measures were collected during the COVID-19 pandemic, which could affect generalizability to other time periods.

## Conclusions

This cohort study of Swedish university students suggests that procrastination is associated with subsequent mental health problems, disabling pain, unhealthy lifestyle behaviors, and worse psychosocial health factors. Considering that procrastination is prevalent among university students, these findings may be of importance to enhace the understanding of students’ health.

## Notes

Supplement 1. **eMethods 1.** Assessment of the Outcome and Confounder Variables **eTable 1.** Comparisons of Estimates Under Different Adjustments **eTable 2.** Characteristics of Participants at Pre-baseline Stratified by Missingness at the Nine-Month Follow-up **eMethods 2.** Sensitivity Analysis Controlling for Prior Levels of Procrastination **eTable 3.** Procrastination (T3) and Subsequent Health Outcomes Six Months Later (T5), Adjusted for Prior Levels of Procrastination (T2) **eMethods 3.** Sensitivity Analysis for the Imputation of the Three Missing Items on the Pittsburgh Sleep Quality Inventory
eReferences

Supplement 2.
Data Sharing Statement
