# Supplementary material for: Associations Between Procrastination and Subsequent Health Outcomes Among University Students in Sweden
Source: JAMA Netw Open. 2023 Jan 4;6(1):e2249346. doi: 10.1001/jamanetworkopen.2022.49346 (PMC9857662; doi:10.1001/jamanetworkopen.2022.49346)
Supplement: Supplement 1. — eMethods 1. Assessment of the Outcome and Confounder Variables eTable 1. Comparisons of Estimates Under Different Adjustments eTable 2. Characteristics of Participants at Pre-baseline Stratified by Missingness at the Nine-Month Follow-up eMethods 2. Sensitivity Analysis Controlling for Prior Levels of Procrastination eTable 3. Procrastination (T3) and Subsequent Health Outcomes Six Months Later (T5), Adjusted for Prior Levels of Procrastination (T2) eMethods 3. Sensitivity Analysis for the Imputation of the Three Missing Items on the Pittsburgh Sleep Quality Inventory eReferences [file jamanetwopen-e2249346-s001.pdf]

Supplementary Online Content

Johansson F, Rozental A, Edlund K, et al. Associations between procrastination and subsequent health outcomes among university students in Sweden. *JAMA Netw Open.* 2023;6(1):e2249346. doi:10.1001/jamanetworkopen.2022.49346

eMethods 1. Assessment of the Outcome and Confounder Variables

eTable 1. Comparisons of Estimates Under Different Adjustments

eTable 2. Characteristics of Participants at Pre-baseline Stratified by Missingness at the Nine-Month Follow-up

eMethods 2. Sensitivity Analysis Controlling for Prior Levels of Procrastination

eTable 3. Procrastination (T3) and Subsequent Health Outcomes Six Months Later (T5), Adjusted for Prior Levels of Procrastination (T2)

eMethods 3. Sensitivity Analysis for the Imputation of the Three Missing Items on the Pittsburgh Sleep Quality Inventory

eReferences

This supplementary material has been provided by the authors to give readers additional information about their work.

© 2023 Johansson F et al. *JAMA Network Open.*

eMethods 1. Assessment of the Outcome and Confounder Variables

Outcomes

Depression, anxiety, and stress symptoms were measured using the short-form Depression, Anxiety and Stress Scale (DASS-21) [1]. DASS-21 consists of 21 items rated from 0 ('did not apply to me at all') to 3 ('applied to me very much, or most of the time') with a one week recall time. These items are divided into the three subscales Depression, Anxiety and Stress, containing seven items each. Summing the items of each subscale gives subscale scores ranging 0-21. DASS-21 has shown good psychometric properties in Swedish university students [2]. Cronbach's alpha was 0.91, 0.79 and 0.86 for the depression, anxiety, and stress subscales respectively at pre-baseline in our sample.

Disabling pain in the neck/upper back, low back, upper extremities and lower extremities were measured using items from the Nordic Musculoskeletal Questionnaire (NMQ) [3], assessing if the participants had at any time during the past three months been prevented from performing their normal activities due to pain in different locations (yes, no). Disabling pain in neck/upper back was defined as disabling pain in neck and/or upper back. Disabling pain in upper extremities was defined as disabling pain in shoulders, elbows and/or hands and disabling pain in the lower extremities defined as disabling pain in the hips, knees, and/or feet. The NMQ has shown good to moderate reliability in general population samples when compared to clinical interviews [4].

Physical inactivity was measured using two items developed by the Swedish National Board of Health and Welfare [5]. The items assess weekly time in physical activity in (1) daily activities and (2) exercise. Physical activity in exercise was multiplied by two and added to the minutes of physical activity in daily activities. Less than 300 minutes of total physical activity per week was considered as being physically inactive, in accordance with recommendations from the World Health Organization [6].

Daily alcohol use was measured using item for frequency of alcohol use from The Alcohol, Smoking and Substance Involvement Screening Test (ASSIST). The recall time was three months and daily alcohol use was defined as using alcohol daily or almost daily [7].

Weekly tobacco use was assessed using the item for frequency of tobacco use from the ASSIST [7], with a recall time of three months. Weekly tobacco used was defined as using tobacco products at least weekly. We chose to focus on weekly, rather than daily, tobacco use, since even non-daily tobacco use show associations to adverse health outcomes [8].

Monthly cannabis use was assessed using the item for frequency of cannabis use from the ASSIST [7] with a recall time of three months. Monthly cannabis use was defined as using cannabis at least monthly.

Breakfast skipping was assessed using the item "How many days a week do you eat breakfast?", with response alternatives 0-7 days a week. Eating breakfast less than five days a week was considered as breakfast skipping.

Poor sleep quality was measured using the Pittsburgh Sleep Quality Index [9] (PSQI). The PSQI is a self-rated measure of sleep quality comprised of 19 items, of which 15 are rated on a scale 0-3 and four items are open ended and recoded to a scale 0-3. The recall time is 30 days. These items are converted to seven different domains, each scored 0-3, representing different aspects of sleep. Summing all domain scores gives a global score ranging 0-21. The PSQI has shown good validity and reliability for measuring sleep quality in university students [10] . In line with previous research a global score of >5 was considered as poor sleep quality.

Loneliness was measured using the UCLA three item loneliness scale [11]. The scale consists of three items rated 1 (Hardly ever) to 3 (Often) which are summed to give a score from 3-9. This short version has shown a high correlation (r = 0.82) with the 20-item revised UCLA loneliness scale [11]. In accordance with previous research loneliness was defined as a score of ≥ 6 [12].

Economic difficulties were assessed using the item "During the last 12 months, have you ever had difficulty in managing the regular expenses for food, rent, bills etc.?", with response categories "No", "Yes, once" and "Yes, more than once". The two latter response categories were considered as having economic difficulties.

Poor general health was assessed with the item "How would you describe your health in general?" with responses ranging from "Very good" to "Very poor" on a five-point scale from. The response categories "Very poor" and "Poor" were considered poor general health.

© 2023 Johansson F et al. *JAMA Network Open.*

Confounders

Age was assessed by asking participants to state their age in years and treated as a continuous variable in the analyses.

Gender was assessed by the question “How do you define your gender identity?”, with response categories “Female”, “Male”, “Other” and “Do not want to identify with either of these identities”. In our analyses the two latter two response categories were collapsed into the category “Other”, due to few responses in these two categories.

Highest parental level of education was assessed by the questions “What is your mother's/father's (parent 1) highest level of education completed?” and “What is your mother's/father's (parent 2) highest level of education completed?”. Response categories were “No formal education” “Less than primary and secondary school (< 9 years)”, “Primary and secondary education (9 years)”, “Upper secondary education (or similar)”, “University (bachelor's or master's)”, “University (doctoral education)” and “Do not know”. We created a binary variable which was coded as “University” if the response categories “University (bachelor's or master's)” or “University (doctoral education)” were selected at any of the questions and coded as “Below university” otherwise.

Previous physical and psychiatric diagnoses was assessed by the question “Have you received any of the following diagnoses from a physician or other care professional?” followed by a list including “rheumatic diseases”, “allergies”, “respiratory diseases”, “cardiovascular diseases”, “gastrointestinal diseases”, “diabetes”, “diseases in urinary tracts or internal or external genitals”, “diseases in the nervous system, eyes or ears”, “mental disorders”, “tumours or cancers” and, “attention deficits or learning disabilities”. Participants responded by indicating “Yes” or “No” for each of the listed diagnoses respectively. A variable indicating the number of previous diagnoses was created by summing all “Yes” responses and treated as a continuous variable in the analyses.

Civil status was assessed by the question “What is your civil status?” with the response categories “Unmarried/single”, “Married/partner (living together)”, “Partner (not living together)”, “Divorced/separated” and, “Widowed”. The response categories “Unmarried/single”, “Divorced/separated” and, “Widowed” were collapsed into a category coded as “Single”. “Married/partner (living together)” was relabelled as “Co-habiting partner” and “Partner (not living together)” was relabelled as “Non-cohabiting partner”. Giving a variable containing the three categories: “Single”, “Co-habiting partner”, and “Non-cohabiting partner”.

Place of birth was assessed by the question “In which country were you born?” with the response categories “Sweden”, “The Nordic countries (Norway, Denmark, Finland, Iceland)”, “Europe (excluding the Nordic countries)” and “Outside Europe”. The labels of the response categories were shortened for ease of presentation.

University education type was assessed by the question “At which university are you studying?” and “At which program are you studying” and recoded manually by the first author to represent the type of education offered by the specific university and education program, into the categories “Technical”, “Medical/health”, “Social science/humanities” or, “Other”.

© 2023 Johansson F et al. *JAMA Network Open.*

**eTable 1.** Comparisons of Estimates Under Different Adjustments [a]			
	**Unadjusted**	**Adjusted for outcome at pre-baseline [b]**	**Fully adjusted [c]**
**Outcome**	Beta/RR (95 % CI)	Beta/RR (95 % CI)	Beta/RR (95 % CI)
Mental health			
Depression symptoms	0.32 (0.29 to 0.36)	0.15 (0.12 to 0.19)	0.13 (0.09 to 0.17)
Anxiety symptoms	0.24 (0.20 to 0.27)	0.11 (0.08 to 0.15)	0.08 (0.04 to 0.12)
Stress symptoms	0.27 (0.23 to 0.31)	0.15 (0.11 to 0.18)	0.11 (0.08 to 0.15)
Disabling pain			
Neck or upper back	1.24 (1.10 to 1.39)	1.14 (1.02 to 1.27)	1.09 (0.96 to 1.24)
Lower back	1.19 (1.05 to 1.34)	1.11 (0.99 to 1.26)	1.03 (0.90 to 1.18)
Upper extremities	1.43 (1.29 to 1.58)	1.34 (1.21 to 1.48)	1.27 (1.14 to 1.42)
Lower extremities	1.19 (1.07 to 1.31)	1.13 (1.02 to 1.24)	1.10 (0.99 to 1.23)
Health behaviours			
Poor sleep quality	1.32 (1.27 to 1.37)	1.16 (1.12 to 1.20)	1.09 (1.05 to 1.14)
Physical inactivity	1.16 (1.12 to 1.20)	1.09 (1.05 to 1.12)	1.07 (1.04 to 1.11)
Daily alcohol use	1.20 (0.89 to 1.62)	1.15 (0.84 to 1.56)	1.05 (0.71 to 1.56)
Weekly tobacco use	1.11 (1.01 to 1.22)	1.01 (0.95 to 1.08)	0.96 (0.89 to 1.04)
Monthly cannabis use	1.58 (1.20 to 2.08)	1.32 (1.04 to 1.69)	1.21 (0.90 to 1.62)
Breakfast skipping	1.26 (1.18 to 1.34)	1.09 (1.03 to 1.15)	1.03 (0.97 to 1.09)
Psycho-social health factors			
Loneliness	1.32 (1.26 to 1.38)	1.13 (1.08 to 1.18)	1.07 (1.02 to 1.12)
Economic difficulties	1.43 (1.28 to 1.59)	1.28 (1.15 to 1.43)	1.15 (1.02 to 1.30)
Poor general health	1.60 (1.39 to 1.84)	1.50 (1.29 to 1.74)	1.14 (0.97 to 1.34)

[a] Procrastination and all continuous outcomes were standardized (mean = 0 and SD = 1) and Beta is the standardized effect size.
[b] Adjusted for the respective outcomes at pre-baseline
[c] Adjusted for the full covariate set (these are the same estimates as presented in Table 2).

© 2023 Johansson F et al. *JAMA Network Open.*

**eTable 2.** Characteristics of Participants at Pre-baseline Stratified by Missingness at the Nine-Month Follow-up [a]		
**Participant characteristics**	**Non-missing at 9 months** (n = 2587)	**Missing at 9 months** (n = 938)
Procrastination, M (SD) [b]	12.8 (5.38)	13.3 (5.55)
Age, M (SD)	24.9 (6.4)	24.5 (5.8)
Gender		
Female, n (%)	1621 (63%)	608 (65%)
Male, n (%) [c]	-	-
Other, n (%) [c]	-	-
Education type		
Medical, n (%)	1193 (46%)	495 (53%)
Technical, n (%)	1101 (43%)	306 (33%)
Social science/Humanities, n (%)	215 (8%)	79 (8%)
Economic, n (%)	46 (2%)	39 (4%)
Other, n (%)	32 (1%)	19 (2%)
Civil status		
Single, n (%)	1452 (56%)	523 (56%)
Cohabiting Partner, n (%)	818 (32%)	315 (34%)
Non-cohabiting Partner, n (%)	317 (12%)	100 (11%)
Place of birth		
Sweden, n (%)	2096 (81%)	730 (78%)
Nordic countries, n (%)	85 (3%)	33 (4%)
Europe, n (%)	141 (5%)	51 (5%)
Outside Europe, n (%)	265 (10%)	124 (13%)
Highest parental education level		
University, n (%)	1901 (73%)	652 (70%)
Below university, n (%)	686 (27%)	286 (30%)
No. previous diagnoses, M (SD)	1.0 (1.2)	1.1 (1.3)
Mental health		
Depression score, M (SD)	4.6 (4.7)	4.9 (4.6)
Anxiety score, M (SD)	2.9 (3.3)	3.3 (3.6)
Stress score, M (SD)	6.2 (4.6)	6.6 (4.5)
Disabling pain		
Neck or upper back, n (%)	278 (11%)	127 (14%)
Lower back, n (%)	262 (10%)	102 (11%)
Upper extremities, n (%)	319 (12%)	117 (12%)
Lower extremities, n (%)	377 (15%)	168 (18%)
Lifestyle behaviours		
Poor sleep quality, n (%)	1339 (52%)	554 (59%)
Physically inactive, n (%)	1330 (51%)	501 (53%)
Daily alcohol use, n (%)	37 (1%)	14 (1%)
Weekly tobacco use, n (%)	380 (15%)	162 (17%)
Monthly cannabis use, n (%)	49 (2%)	23 (2%)
Breakfast skipping, n (%)	595 (23%)	283 (30%)
Psycho-social health factors		
Loneliness, n (%)	1081 (42%)	404 (43%)
Economic difficulties, n (%)	353 (14%)	202 (22%)
Poor general health, n (%)	87 (3%)	26 (3%)

[a] Percentages of some categorical variables do not add up to 100 % due to rounding.

© 2023 Johansson F et al. *JAMA Network Open.*

[b] Procrastination was measured at baseline, while all covariates and outcomes were measured at pre-baseline.
[c] The gender categories "Male" and "Other" are not shown due to low cell counts in the category "Other".

© 2023 Johansson F et al. *JAMA Network Open.*

eMethods 2. Sensitivity Analysis Controlling for Prior Levels of Procrastination

It has been recommended that outcome-wide studies should also control for pre-baseline levels of exposures [13]. By adjusting for previous levels of the exposures, the estimates can be seen as referring to "incident" rather than "prevalent" exposure, and the risk of reverse causation and unmeasured confounding is limited [13]. However, due to the high correlation between procrastination at different time-points in our sample (Pearson's *r* was 0.79 over three months) we decided not to control for prior levels of procrastination in our main analyses to avoid multicollinearity.

Further, in our data collection, procrastination was not measured at the pre-baseline time-point (T1 in Figure 1). To be able to control for prior procrastination levels, we estimated the effect of procrastination measured three months after baseline (T3 in Figure 1), while controlling for procrastination levels at baseline (T2 in Figure 1). We used the same outcomes and covariates, measured at the same time-points as in the main analysis. However, since the exposure measurement was shifted three months forward as compared to the main analysis, the outcomes in this sensitivity analysis were measured six, rather than nine, months after exposure. The analytic sample for these analyses included the 2479 participants, who responded at time-points T1, T2, T3 and T5 (Figure 1). The results are presented in eTable 3 below, showing that estimates were similar to those of the main analysis.

© 2023 Johansson F et al. *JAMA Network Open.*

eTable 3. Procrastination (T3) and Subsequent Health Outcomes Six Months Later (T5), Adjusted for Prior Levels of Procrastination (T2) [a, b]

Outcome	Beta	RR	95 % CI
Mental health			
Depression symptoms	0.12	-	0.07 to 0.17
Anxiety symptoms	0.09	-	0.03 to 0.14
Stress symptoms	0.11	-	0.06 to 0.17
Disabling pain			
Neck or upper back	-	1.07	0.88 to 1.31
Lower back	-	1.02	0.84 to 1.24
Upper extremities	-	1.14	0.97 to 1.34
Lower extremities	-	0.99	0.84 to 1.17
Health behaviours			
Poor sleep quality	-	1.18	1.11 to 1.25
Physical inactivity	-	1.03	0.98 to 1.09
Daily alcohol use	-	1.12	0.71 to 1.77
Weekly tobacco use	-	1.06	0.96 to 1.18
Monthly cannabis use	-	1.85	1.08 to 3.16
Breakfast skipping	-	1.07	0.97 to 1.18
Psycho-social health factors			
Loneliness	-	1.12	1.04 to 1.20
Economic difficulties	-	1.40	1.16 to 1.69
Poor general health	-	1.19	0.92 to 1.55

[a] Procrastination and all continuous outcomes were standardized (mean = 0 and SD = 1) and Beta is the standardized effect size.
[b] All models were adjusted for the set of pre-baseline covariates described in the main article, including all outcomes at pre-baseline and procrastination at baseline.
CI = Confidence interval, DP = Disabling pain, LCL = Lower confidence limit, PE = Point estimate, RR = Relative risk.

© 2023 Johansson F et al. *JAMA Network Open.*

eMethods 3. Sensitivity Analysis for the Imputation of the Three Missing Items on the Pittsburgh Sleep Quality Inventory

Three items of the sleep disturbances component of the PSQI were missing for 225 participants in the analytic sample, due to a technical error at the beginning of data collection (item 5b, 5f and 5j). In the main analyses, this was handled by imputing the person-mean of the remaining items of the sleep disturbances component (5b-j) of the PSQI. As a sensitivity analysis, we also performed multiple imputation (MI) of the three missing items. Analyses were performed using the *mice* package in R. We used chained equations to create five imputed datasets. All covariates used in the main outcome models, as well as the individual items of the PSQI at pre-baseline and follow-up were used to predict missing values with predictive mean matching. We only imputed the three missing items (5b, 5f and 5j), for participants who were not lost to follow-up.

We then ran the outcome model for poor sleep quality and pooled the results from the five imputed datasets, using the same covariates as in the main model. A one SD increase in procrastination at baseline was associated to an adjusted RR of poor sleep quality of 1.09 (95% CI: 1.05 – 1.14) at the nine-month follow-up, and a crude RR of 1.30 (95 % CI: 1.25 – 1.36). The adjusted RR is identical to that of the main analysis using person-mean imputation, when rounded to the second decimal (Table 2). The crude RR is also very close to the estimates obtained using person-mean imputation (eTable 1).

Further, we also ran the outcome model for poor sleep quality while excluding the 225 participants with missing items on the PSQI. This again gave an adjusted RR of 1.09 (95% CI: 1.05 – 1.14).

© 2023 Johansson F et al. *JAMA Network Open.*

eReferences

1. Henry JD, Crawford JR. The short-form version of the Depression Anxiety Stress Scales (DASS-21): construct validity and normative data in a large non-clinical sample. *Br J Clin Psychol.* 2005;44(Pt 2):227-239.
2. Alfonsson S, Wallin E, Maathz P. Factor structure and validity of the Depression, Anxiety and Stress Scale-21 in Swedish translation. *J Psychiatr Ment Health Nurs.* 2017;24(2-3):154-162.
3. Kuorinka I, Jonsson B, Kilbom A, et al. Standardised Nordic questionnaires for the analysis of musculoskeletal symptoms. *Appl Ergon.* 1987;18(3):233-237.
4. Dahl AG, Havang S, Hagen K. Reliability of a self-administrated musculoskeletal questionnaire: The fourth Trøndelag health study. *Musculoskeletal Science and Practice.* 2022;57:102496.
5. Socialstyrelsen. Nationella riktlinjer för sjukdomsförebyggande metoder 2011. Bilaga: Indikatorer. 2011.
6. Bull FC, Al-Ansari SS, Biddle S, et al. World Health Organization 2020 guidelines on physical activity and sedentary behaviour. *Br J Sports Med.* 2020;54(24):1451-1462.
7. Humeniuk R, Ali R, Babor TF, et al. Validation of the Alcohol, Smoking And Substance Involvement Screening Test (ASSIST). *Addiction.* 2008;103(6):1039-1047.
8. Inoue-Choi M, Christensen CH, Rostron BL, et al. Dose-Response Association of Low-Intensity and Nondaily Smoking With Mortality in the United States. *JAMA Network Open.* 2020;3(6):e206436-e206436.
9. Buysse DJ, Reynolds CF, 3rd, Monk TH, Berman SR, Kupfer DJ. The Pittsburgh Sleep Quality Index: a new instrument for psychiatric practice and research. *Psychiatry Res.* 1989;28(2):193-213.
10. Liu D, Kahathuduwa C, Vazsonyi AT. The Pittsburgh Sleep Quality Index (PSQI): Psychometric and clinical risk score applications among college students. *Psychol Assess.* 2021;33(9):816-826.
11. Hughes ME, Waite LJ, Hawkley LC, Cacioppo JT. A Short Scale for Measuring Loneliness in Large Surveys: Results From Two Population-Based Studies. *Res Aging.* 2004;26(6):655-672.
12. Steptoe A, Shankar A, Demakakos P, Wardle J. Social isolation, loneliness, and all-cause mortality in older men and women. *Proc Natl Acad Sci U S A.* 2013;110(15):5797-5801.
13. VanderWeele TJ, Jackson JW, Li S. Causal inference and longitudinal data: a case study of religion and mental health. *Soc Psychiatry Psychiatr Epidemiol.* 2016;51(11):1457-1466.

© 2023 Johansson F et al. *JAMA Network Open.*
