# Supplementary material for: Associations Between Procrastination and Subsequent Health Outcomes Among University Students in Sweden
Source: JAMA Netw Open. 2023 Jan 4;6(1):e2249346. doi: 10.1001/jamanetworkopen.2022.49346 (PMC9857662; doi:10.1001/jamanetworkopen.2022.49346)
Supplement: Supplement 2. — Data Sharing Statement [file jamanetwopen-e2249346-s002.pdf]

Data Sharing Statement

Johansson. Associations Between Procrastination and Subsequent Health Outcomes Among University Students in Sweden. *JAMA Netw Open*. Published January 04, 2023. doi:10.1001/jamanetworkopen.2022.49346

Data
Data available: No

Additional Information
Explanation for why data not available: The dataset analysed in the current study is not publicly available due to secondary confidentiality and privacy of the participants.
